# Cultivation Renaissance in the Post-Metagenomics Era: Combining the New and Old

**DOI:** 10.1264/jsme2.ME3402rh

**Published:** 2019-06-30

**Authors:** Hideyuki Tamaki

**Affiliations:** 1 Bioproduction Research Institute, National Institute of Advanced Industrial Science and Technology (AIST) Tsukuba Central 6, Tsukuba, Ibaraki 305–8566 Japan

Since the era of Robert Koch, cultivation has been the most fundamental microbiological method to examine the taxonomy, morphology, physiology, metabolism, and positive and negative functions of microorganisms inhabiting humans, animals, plants, and other artificial and natural ecosystems. Cultivation is a key method in many fields of biology and industry; it is sometimes used to identify pathogens and develop counter-measures, develop medicines, functional foods, and various materials, such as biodegradable plastics, and even elucidate the nature of microorganisms in various natural environments. However, advent of molecular biological approach revealed one of the most surprising discoveries in microbiology—the overwhelming diversity and abundance of uncultured microorganisms across Earth’s environments. The following leap in technology, namely, next-generation sequencing (NGS) and “omics” tools, have recently enabled researchers to investigate the genomic compositions of these microorganisms and elucidate their functions (10). However, precise interpretations remain challenging due to large gaps in knowledge and corresponding between cultured organisms, characterized genes, and the currently increasing number of genomes of the uncultured (35). To address this issue, researchers are beginning to revisit cultivation-based microbiology (4, 11, 24, 43, 51).

One of the most important achievements led by the NGS-based approach was to uncover the world of the human microbiome. This approach has revealed the composition, diversity, distribution, genomic traits, and metabolic capacities of human-associated microorganisms, and also implicated the human microbiome in a wide variety of diseases, including lifestyle diseases (*e.g*., obesity and type 2 diabetes), ulcerative colitis, Crohn’s disease, non-alcoholic steatohepatitis, arteriosclerosis, nephritis, rheumatoid arthritis, atopic dermatitis, Parkinson’s disease, and even metal illnesses (*e.g*., clinical depression) (1, 9, 12, 20, 36). This has attracted worldwide interest due to the potential impact of human-associated microorganisms in the field of biological sciences, including microbial ecology, and even in medical and healthcare industries. However, specific microorganisms driving disease have rarely been identified and characterized due to the lack of axenic cultures of the possible causative agent. Kameyama and co-workers discovered and characterized a novel intestinal bacterium involved in the development of type 2 diabetes in mice (13, 16). They succeeded in isolating this unique bacterium from the feces of hyperglycemic obese mice. When inoculated into germ-free (GF) ob/ob mice, the isolate colonized GF mice and induced the typical symptoms of diabetes, such as significant increases in fasting blood glucose levels as well as liver and mesenteric adipose tissue weights and decreases in plasma insulin levels and HOMA-β values. Kusada *et al*. further revealed that this isolate was phylogenetically novel at least at the genus level in the family *Lachnospiraceae* (the phylum *Firmicutes*), and proposed a new taxonomic name, *Fusimonas intestini* gen. nov., sp. nov. for the new strain with morphological and physiological characterization (16). In 2012, Qin *et al*. reported that a population of uncultured members of *Lachnospiraceae* in the intestines positively correlated with type 2 diabetes, suggesting that *Lachnospiraceae* bacteria might be related to diabetes; however, no direct evidence was provided (40). Cultivation demonstrated for the first time that intestinal *Lachnospiraceae* members could involve in development of the disease.

Antibiotic resistance is also of great interest because its recent misuse and overuse have led to the worldwide emergence of multidrug-resistant pathogens that pose a serious threat in the clinical, agriculture, aquaculture, and livestock fields. Nevertheless, very little is known about the mechanisms underlying the occurrence and extensive distribution of antibiotic-resistant pathogens. In *Microbes and Environments*, recent studies reported the occurrence and distribution of plausible pathogenic bacteria (*e.g*., *Escherichia coli*, *Enterococcus* sp., *Salmonella* sp., and *Mycobacterium* sp.) as well as their antibiotic resistance capabilities and genetic elements through the extensive cultivation and isolation of the targeted organisms (2, 5, 25, 34, 42, 50). For example, Chirila *et al*. investigated *E. coli* isolated from young animals between 1980–2016, and revealed apparent increases in antibiotic resistance in pathogenic *E. coli* producing shiga toxin (5). Antibiotic resistance in *E. coli* was also reported in water environments in northern Thailand (2), neonatal calves in Uruguay (50), and pigs from an intensive production farm in Poland (25).

Kusada *et al*. recently isolated a novel multidrug-resistant bacterium, *Acidovorax* sp. strain MR-S7 from activated sludge in a penicillin G production wastewater treatment process (17, 54). In examinations of this new strain, they unexpectedly found a very unique antibiotic-resistant enzyme (MacQ) that is a bifunctional acylase capable of degrading not only multiple β-lactam antibiotics, but also various *N*-acylhomoserine lactone (AHL) derivatives, which are known to be signal molecules for bacterial cell-cell communication (quorum sensing). *Acidovorax* sp. strain MR-S7 is highly resistant to multiple β-lactam antibiotics and exhibits obvious quorum-quenching activity to inhibit bacterial cell-cell communication. The same research group succeeded in isolating multiple β-lactam antibiotic-resistant bacteria with quorum-quenching activities (18). This is the first discovery of a hitherto-overlooked antibiotic resistance mechanism mediated by quorum-quenching enzymes through extensive cultivation followed by genomic, genetic, and biochemical analyses. These unexpected findings were the first to link two biological functions, antibiotic resistance and quorum quenching, which have long been thought to be distinct, and further infer a functional and evolutionary correlation between them.

Plant-associated microorganisms have been extensively investigated because of their impact on both academia and industries relevant to the fields of agriculture, food, chemical, energy, and the environment. Recent publications in *Microbes and Environments* provided interesting findings related to plant-associated bacteria and fungi. Boudjeko *et al*. isolated and characterized a novel species of actinomycetes, *Streptomyces cameroonensis* sp. nov., from the rhizosphere of a (sub)tropical sunflower species, *Chromolaena odorata*, in Cameroon. They found that the novel isolate produced geldanamycin with antibacterial and antifungal activities, and further demonstrated its ability to promote the growth of a cacao plant (*Theobroma cacao*) (3). Bustillos-Cristales *et al*. cultivated and isolated a wide variety of methylotrophic bacteria from the rhizosphere, plant surface, and inside the stem of *Neobuxbaumia macrocephala*, a long-lived columnar cactus, and suggested a relationship between rare-earth element-dependent XoxF methanol dehydrogenases found in the isolates and their adaptability to xerophytic plants (6). Fungi-bacteria interactions (*e.g*., *Rhizobium* sp.-endophytic fungi, soil indigenous bacteria-pathogenic *Fusarium* sp., and *Burkholderiaceae-*related endobacteria and plant and soil fungi *Mortierella* spp.) related to plant growth and health were also investigated (8, 26, 44). Mourad *et al*. modified the previously proposed culturing method using plant only-based culture media for plant-associated rhizobacteria. They sought more suitable plant materials for media, and demonstrated that newly tailored artificial media led to the cultivation of *in situ* rhizobacteria of barley, even endophytes; however, further modifications are needed for the cultivation of difficult-to-culture bacteria (28).

The body of aquatic plants (the root, frond, and leaf) appears to be an attractive source for the cultivation and isolation of yet-to-be cultured bacteria recognized as “fastidious” bacteria (23, 45, 47). Tanaka *et al*. investigated aquatic plant-associated bacteria and succeeded in isolating a number of phylogenetically novel bacteria from the roots of two emerging plants, *Iris pseudacorus and Scirpus juncoides* (48). They found that 42–47% of the isolates (a total of 280 strains) were phylogenetically novel based on 16S rRNA gene sequences, and obtained 17 and 8 pure isolates of the phyla *Acidobacteria* and *Verrucomicrobia*, respectively, which had been widely known as rarely cultured bacterial taxa. Interestingly, the success of this isolation did not require any advanced technology or specific factors; it merely involved simple cultivation using low-nutrient agar plates and long-term incubations. Bacteria inhabiting aquatic plant bodies are likely active and, thus, more prone to be cultivated than in other environments, such as soils and waters. Based on this and previous findings (47), the same research group developed a new method for the cultivation of uncultured microorganisms, called the “Duckweed-Microbe Co-Cultivation Method” (49). This concept involves the use of aseptic duckweed as a fishing hook for the capture and enrichment of yet-to-be cultured microorganisms from environments. A co-culture of aseptic duckweed and microbial cells harvested from environmental samples (*Japanese loosestrife* in the study), followed by conventional agar plate cultivation, allowed a wide variety of novel microorganisms to be isolated, including two novel strains of the rarely cultivated phylum *Armatimonadetes* (formally called candidate division OP10). The duckweed-microbe co-cultivation method may enable pure cultures of yet-to-be cultured microorganisms to be obtained in various environments as well as aquatic plants keeping unique uncharacterized microbial taxa.

Deep subsurface microorganisms and their ecophysiologies and lifestyles still remain largely unknown (7, 15, 27, 29, 30, 32, 33, 38, 39, 46, 53). By using geochemical analyses, cultivation, and molecular-based approaches, Matsushita *et al*. provided evidence of microbial methane production in deep aquifers of the accretionary prism (22), and also found regional variations in methane and nitrogen production processes in these ecosystems (21). Kato *et al*. investigated microorganisms in deep subsurface high temperature oil reservoirs and demonstrated that conductive metal materials (iron oxides) promoted the methanogenic acetate degradation of subsurface communities using elaborate cultivation experiments (15). Nishihara *et al*. successfully isolated novel thermophilic chemolithoautotrophic bacteria in the phylum *Aquificae* from a hot spring aquifer, and was the first to demonstrate the nitrogenase (nitrogen fixation) activity of these thermophiles (37). Imachi *et al*. successfully cultured microorganisms inhabiting 2-km-deep, 20-million-year-old subseafloor coalbeds using long-term bioreactor cultivation (more than 1,000 d), and revealed that the community was associated with methanogenic lignite-derived organic matter degradation (11). Nakahara *et al*. isolated an unprecedentedly fastidious slow-growing bacterium from a deep subseafloor sediment microbial community cultivated in a continuous flow bioreactor, revealed its ecophysiology, and proposed a new genus and species name, *Aggregatilinea lenta*, for this strain, a representative for the new order *Aggregatilineales* of the class *Anaerolinea* (phylum *Chloroflexi*) (31).

Mayumi *et al*. discovered “a coal-eating methanogen” from deep subsurface environments (24). This organism is the *Methermicoccus shengliensis* strain AmaM isolated from deep subsurface oil reservoirs, and was proven to directly form methane from coals with different maturities. The authors also revealed that this deep subsurface-derived methanogen had the ability to produce methane from more than 30 types of methoxylated aromatic compounds (MACs, R-OCH_3_) known to be lignin-derived compounds and abundant in coals. This is a very important finding because textbooks worldwide state that methanogens utilize a very limited range of substrates: only three types of simple compounds (*i.e*., H_2_/CO_2_, acetate, and methyl compounds, such as methanol and methylamines). More than 150 species of methanogens have been obtained over the past 50 years; however, no methanogenic capability from unexpectedly complex compounds, such as MACs and coals, was found. Although the whole metabolic pathway of MAC-driven methanogenesis has not yet been elucidated in detail, genomic analyses and isotope tracer experiments using pure cultures inferred a novel mode of methanogenesis, “methoxydotrophic methanogenesis”, the metabolic pathway of which is significantly distinct from the three other pathways of hydrogenotrophic, acetoclastic, and methylotrophic methanogenesis. The discovery of a novel subsurface methanogenic niche, “methoxydotrophic methanogenesis”, changes our perception of methanogens and their ecophysiology and roles in the global carbon cycle, and further suggests that a hitherto unforeseen route to methane from MACs contributes to the formation of natural gas resources, including coalbed methane, known as one of the largest unconventional natural gas resources in the world.

“These exciting findings show that, even in the metagenomics era, cultivation of fastidious microorganisms is necessary to discover novel types of metabolism.”

—Cornelia U. Welte, Insights/Perspective, Science 354: 184 (2016) (52)

The discovery of an intestinal diabetes-inducing bacterium, novel aquatic plant growth-promoting bacteria, and a deep subsurface coal-eating methanogen could not have been brought by conventional omics approaches without cultivation because unforeseen biological phenotypes have not yet been linked to genetic components. However, this does not discount the potential of omics. The combined use of cultivation and metagenomics recently revealed uncultured syntrophic ethane/butane-degrading archaea (4, 19), syntrophic alkane-degrading bacteria (41), and nitrogen-cycling thermophiles in subsurface environments (14). Cultivation-based studies have potential risks of failure in research. Target organisms may not grow and, even when they do, maintaining the cultures is highly challenging; more importantly, this work is highly dependent on a scientist’s intuition. As referred to in a *Nature* News Feature, Professor Schleper commented on her endeavor to cultivate the mysterious uncultured Asgard archaea: “[this is] *the craziest project I’ve ever applied for money for*” (51). Such craziness is paramount for culturing these fastidious microorganisms. Time goes around—the cultivation renaissance has arrived.
